# Impact of Percutaneous Mitral Valve Repair Using the MitraClip^TM^ System on Ventricular Arrhythmias and ICD Therapies

**DOI:** 10.3390/life12030344

**Published:** 2022-02-25

**Authors:** Nicolas A. Geis, Anna Göbbel, Michael M. Kreusser, Tobias Täger, Hugo A. Katus, Norbert Frey, Philipp Schlegel, Philip W. Raake

**Affiliations:** Department of Internal Medicine III, University of Heidelberg, Im Neuenheimer Feld 410, 69120 Heidelberg, Germany; anna-goebbel@web.de (A.G.); michael.kreusser@med.uni-heidelberg.de (M.M.K.); tobias.taeger@med.uni-heidelberg.de (T.T.); hugo.katus@med.uni-heidelberg.de (H.A.K.); norbert.frey@med.uni-heidelberg.de (N.F.); philipp.schlegel@med.uni-heidelberg.de (P.S.); philip.raake@med.uni-heidelberg.de (P.W.R.)

**Keywords:** MitraClip^TM^, mitral regurgitation, percutaneous mitral valve repair, ventricular arrhythmia, ICD therapy, transcatheter edge-to-edge repair, TEER

## Abstract

Transcatheter edge-to-edge repair (TEER) using the MitraClip™ device has been established as a suitable alternative to mitral valve surgery in patients with severe mitral regurgitation (MR) and high or prohibitive surgical risk. Only limited information regarding the impact of TEER on ventricular arrhythmias (VA) has been reported. The aim of the present study was to assess the impact of TEER using the MitraClip^TM^ device on the burden of VA and ICD (Implantable Cardioverter Defibrillator) therapies. Among 600 MitraClip^TM^ implantations performed in our clinic between September 2009 and October 2018, we identified 86 patients with successful TEER and an active implantable cardiac device (pacemaker, ICD, CRT-P/D (Cardiac Resynchronization Therapy-Pacemaker/Defibrillator)) eligible for retrospective VA analyses. These patients presented with mainly functional MR (81.4%) and severely reduced left ventricular ejection fraction (mean LVEF 22.1% ± 10.3%). The observation period comprised 456 ± 313 days before and 424 ± 287 days after TEER. The burden of ventricular arrhythmias (sustained ventricular tachycardia (sVT) and ventricular fibrillation (VF)) was significantly reduced after TEER (0.85 ± 3.47 vs. 0.43 ± 2.03 events per patient per month, *p* = 0.01). Furthermore, the rate of ICD therapies (anti-tachycardia pacing (ATP) and ICD shock) decreased significantly after MitraClip^TM^ implantation (1.0 ± 3.87 vs. 0.32 ± 1.41, *p* = 0.014). However, reduction of VA burden did not result in improved two-year survival in this patient cohort with severely reduced LVEF. Mitral valve TEER using the MitraClip™ device was associated with a significant reduction of ventricular arrhythmias and ICD therapies.

## 1. Introduction

Chronic severe mitral regurgitation (MR) results in cardiac remodelling involving left ventricular (LV) and left atrial (LA) enlargement, deterioration of LV contractile function and increased myocardial fibrosis due to chronic volume overload [1,2]. Severe MR is an independent predictor of mortality in heart failure (HF) patients irrespective of ischaemic or non-ischaemic aetiology [3,4,5]. Congestive HF with concomitant severe functional MR is associated with an increased incidence of ventricular arrhythmias (VA) [6,7].

Transcatheter edge-to-edge mitral valve repair (TEER) is an established alternative to mitral valve surgery in patients with severe MR and high or prohibitive surgical risk [8,9,10]. Previous studies have shown reverse cardiac remodelling following TEER including a reduction of LV and LA dimensions as well as an improvement of left ventricular ejection fraction (LVEF) [11]. However, TEER not only resulted in reverse remodelling but also in improvement of clinical endpoints such as a lower rate of HF hospitalisation and lower all-cause mortality in comparison to guideline-directed medical therapy (GDMT) [12].

To date, only limited information regarding the effect of TEER on VA burden has been reported [13,14,15]. Therefore, in the present study we investigated the impact of TEER on VA rates, ICD (Implantable Cardioverter Defibrillator) therapies and two-year survival rate in patients with severe symptomatic MR and HF with reduced ejection fraction (HFrEF).

## 2. Materials and Methods

### 2.1. Patient Population

The study cohort was recruited from 600 patients treated with the MitraClip^TM^ device (Abbott Vascular Devices, Santa Clara, CA, USA) for severe MR in our tertiary centre between September 2009 and October 2018. All patients suffered from symptomatic (New York Heart Association (NYHA) functional class ≥ II) severe MR despite GDMT. The decision to perform TEER and refrain from surgery was the result of an interdisciplinary evaluation of the heart team. Due to our centres heart transplantation programme the study cohort comprises 18 patients (21%, 18/86) enlisted for heart transplantation.

To retrospectively analyse the impact of TEER on VA and ICD therapies, only patients with successful MitraClip^TM^ implantation and an implanted electrical cardiac device (pacemaker, ICD, CRT-P (Cardiac Resynchronization Therapy-Pacemaker) or CRT-D (Cardiac Resynchronization Therapy-Defibrillator)) were included in the study, irrespective of systolic LV-function prior TEER. Arrhythmic events and anti-arrhythmic therapies detected by the implanted electrical devices were analysed within a timeframe of two years before to two years after TEER.

Forty (46.5%) of the included 86 patients had already experienced a sustained ventricular tachycardia (sVT) or ventricular fibrillation (VF) prior to TEER in the analysed timeframe and in 37/80 (46.3%) patients an adequate ICD therapy, mainly anti-tachycardia pacing (ATP), had already occurred.

### 2.2. MitraClip^TM^ Procedure

Procedural characteristics of TEER using the MitraClip^TM^ device have been described in detail previously [11,16]. All procedures were performed under general anaesthesia with fluoroscopic and transoesophageal echocardiographic guidance. In the majority of patients, closure of the femoral vein puncture site was achieved using the Perclose Proglide System (Abbott Vascular Devices, Santa Clara, CA, USA) [17]. In some patients a cutaneous “figure eight” suture was necessary for access site closure. Patients were extubated and transferred to our intermediate care unit for a post-interventional observation period of at least 6 h. All patients received anticoagulation for a minimum of four weeks after intervention, which is the standard treatment in our centre, as described previously [18]. Of note, anticoagulation does not represent the medical treatment commonly used after TEER, which rather consists of dual antiplatelet therapy using aspirin and clopidogrel for 3–6 months. Postprocedural results and clinical adverse events after TEER were reported according to the recommendations of the Mitral Valve Academy Research Consortium (MVARC) [19]. However, device success was measured at the time of hospital discharge.

### 2.3. Echocardiographic Assessment

All patients received baseline echocardiographic and haemodynamic evaluation [10]. The echocardiographic assessment followed an integrative approach according to current recommendations. Severity of MR was graded as none or trace, mild (1), moderate (2) and severe (3) [10,20]. MR aetiology was classified as functional, degenerative and mixed.

### 2.4. Arrhythmia Detection

Analyses of VA events and ICD therapies were performed on the basis of implanted device recordings. In addition, clinical follow-up reports and local databases comprising in-house or remote device Holter information were used. Assessment and classification of VA events were conducted by institutional electrophysiology experts. Ventricular tachycardia (VT) lasting for ≥30 or <30 s was classified as sustained (sVT) or non-sustained (nsVT), respectively. To fulfil nsVT criteria, at least ≥3 consecutive premature ventricular complexes (cycle length < 500 ms) were mandatory. Only appropriate ICD therapies (anti-tachycardia pacing and shocks due to VA) were included in the analyses.

In order to achieve better comparability, VA episodes and ICD therapies were calculated as events per patient per month.

### 2.5. Statistics

Continuous variables were summarised as mean ± standard deviation or median and interquartile range and compared using paired or unpaired Student’s *t*-test.

If normal distribution could not be demonstrated Wilcoxon rank sum test or the Mann–Whitney U test were used. Derangement from normal distribution was assessed using Shapiro-Wilk test. Categorical variables were described as percentages and compared using Chi-square or Fisher’s exact tests, respectively, depending on the expected frequency above or below 5. Paired categorical variables before and after TEER were compared using McNemar’s test. Survival data were summarised by Kaplan-Meier survival curves and unadjusted survival rates were compared using the log rank test. Multivariate Cox regression using stepwise forward selection was performed to analyse the influence of relevant variables on two-year mortality. Univariate regression was used to estimate procedural results.

A two-tailed *p*-value < 0.05 was considered statistically significant. For statistical analysis SPSS Statistics 27 for Microsoft Windows and Stata 16 was used.

Our retrospective study conforms with the principles outlined in the *Declaration of Helsinki*. All patients were informed about the specific risks and alternatives of MitraClip^TM^ therapy and gave informed written consent to the procedure. The study protocol was in accordance with the local ethics committee.

## 3. Results

### 3.1. Patient Characteristics

**Figure 1:**
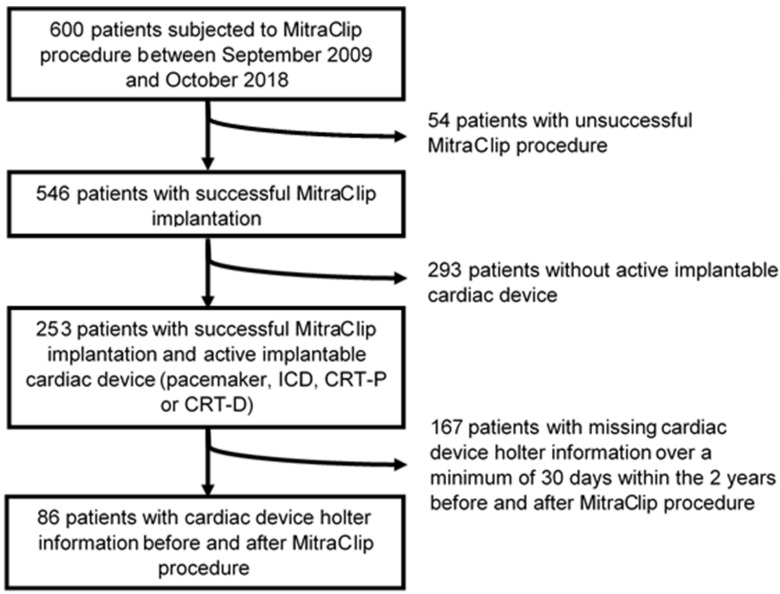
Patient flow chart.

Among 600 patients who were subjected to TEER using the MitraClip^TM^ device between September 2009 and October 2018, 86 fulfilled the aforementioned inclusion criteria (Figure 1).

Median age was 66.5 [58; 76] years and 80.2% (69/86) were male. Predominant pathology of the mitral valve was functional MR (81.4%, 70/86 patients). Mean LVEF of the included patients was severely reduced (LVEF 22.1% ± 10.3%) with 90.7% (78/86) presenting with LVEF ≤ 35%. All patients were severely symptomatic at the time of the procedure with 88.4% (76/86) presenting in NYHA functional class ≥ III.

The advanced stage of disease is also displayed by advanced LV (left ventricular end diastolic diameter (LVEDD) 68.2 mm ± 11.0 mm) and LA dilatation (54.6 mm ± 9.7 mm) as well as pronounced elevation of NT-proBNP-levels (median 4567 ng/l [1957; 10,630]).

A detailed overview of patients’ baseline characteristics is shown in Table 1.

Baseline characteristics and procedural outcome data of the excluded 167 patients with missing cardiac device Holter information are summarised in Supplementary Table S1.

### 3.2. Procedural Outcome

Technical success rate among all patients subjected to TEER in the study period was 91% (546/600). Among the 86 patients with implanted electrical devices suitable for arrhythmic analyses, MitraClip^TM^ device success rate was 97.7% (84/86). In one patient periprocedural partial clip detachment occurred during placement of a second MitraClip^TM^ device, requiring a third MitraClip^TM^ for stabilization and sufficient MR reduction. In another patient an arterio-venous fistula was observed, requiring surgical repair. More than one MitraClip^TM^ was implanted in 31 (36.0%) patients. Only in the above mentioned patient with partial clip detachment were more than two devices necessary.

### 3.3. Arrhythmic Outcomes

The observation period comprised 456 ± 313 days before and 424 ± 287 days after TEER.

### 3.4. Ventricular Arrhythmia

Overall, 61 patients (70.9%) had already experienced at least one VA episode (nsVT, sVT and/or VF) within the analysed study period prior to TEER. In 58.1% (50/86) at least one nsVT, in 41.9% (36/86) at least one sVT and in 12.8% (11/86) one VF episode had already occurred (Table 1). No significant differences in baseline characteristics were found when comparing patients with prior VA episodes and patients without prior VA episodes. However, there was a trend towards more pronounced LV dilatation in patients with prior VA episodes (*p* = 0.08, data not shown).

After TEER, at least one VA occurred in 46 patients (53.5%). At least one nsVT, sVT or VF episode was recorded in 47.7% (41/86), 23.3% (20/86) or 3.5% (3/86) of patients, respectively.

The burden of total VA episodes (nsVT, sVT, VF) was significantly reduced after TEER (2.24 ± 5.09 vs. 1.26 ± 3.52 events per patient per month, *p* = 0.019). While non-sustained VTs were not significantly decreased (1.39 ± 3.31 vs. 0.83 ± 2.09 events per patient per month, *p* = 0.12), the burden of sustained VTs was significantly reduced after TEER (0.82 ± 3.46 vs. 0.43 ± 2.03 events per patient per month, *p* = 0.014). Concerning VF episodes, a non-significant trend for less events could be detected (0.035 ± 0.186 vs. 0.005 ± 0.033 events per patient per month, *p* = 0.056) (Table 2). As a consequence, the overall burden of sustained ventricular arrhythmias (sVT and VF) was also significantly reduced (0.85 ± 3.47 vs. 0.43 ± 2.03 events per patient per month, *p* = 0.01).

### 3.5. ICD Therapies

Overall, 37 patients (46.3%) had already experienced at least one appropriate ICD therapy within the analysed study period prior TEER. In 41.3% (33/80) at least one appropriate anti-tachycardia pacing (ATP) and in 27.5% (22/80) at least one appropriate ICD shock had already occurred.

After TEER at least one appropriate ICD therapy occurred in 21 patients (26.3%). At least one appropriate ATP or ICD shock was recorded in 21.3% (17/80) or 17.5% (14/80) of patients, respectively.

The rate of appropriate ICD therapies (ATP and ICD shock) decreased significantly after TEER (1.0 ± 3.87 vs. 0.32 ± 1.41 events per patient per month, *p* = 0.014). While there was only a non-significant trend towards reduction of appropriate ICD shocks (0.18 ± 0.95 vs. 0.04 ± 0.12 events per patient per month, *p* = 0.052), the burden of appropriate ATPs was significantly reduced after TEER (0.82 ± 3.56 vs. 0.28 ± 1.31 events per patient per month, *p* = 0.008) (Table 2).

Reduction of VA episodes and ICD therapies remained significant even when patients were excluded who received VT ablation, Amiodarone therapy or an upgrade to a CRT device (*n* = 14 patients) during follow-up (data not shown).

### 3.6. Characteristics of Patients with Reduced VA Burden Post TEER

In 44 patients (51.2%) a reduction of VA burden (nsVT, sVT, VF) was observed after TEER. No reduction could be detected in 24 patients (27.9%). The remaining 18 patients (20.9%) experienced no VA episode during the analysed study period, neither prior to nor post MitraClip^TM^-implantation.

Patients presenting with reduced VA burden were in a higher NYHA functional class (3.2 ± 0.5 vs. 2.8 ± 0.6; *p* = 0.004), less frequently suffered from ischaemic cardiomyopathy (28/44 vs. 21/24; *p* = 0.048) and revealed larger LV dimensions (LVEDD 70.3 mm ± 10.6 mm vs. 64.6 mm ± 10.4 mm; *p* = 0.037) prior TEER (Table 3).

### 3.7. Echocardiographic and Clinical Outcomes

**Figure 2:**
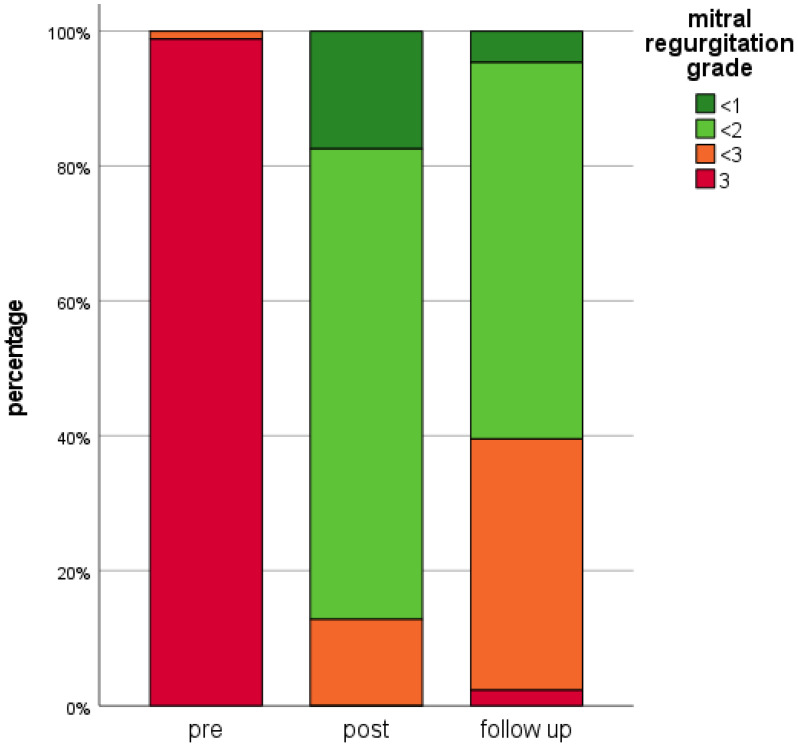
Mitral regurgitation at baseline and follow-up.

In all patients TEER successfully reduced MR by at least one grade post-procedure. At the time point of the last included implantable cardiac device control (mean 424 ± 287 days after MitraClip^TM^-implantation) echocardiographic and clinical data were available for all patients. Median MR grade was 1.5 (1.0; 2.0). Seventy-six patients (88.4%) had an MR grade ≤ 2 and only two patients presented with recurrent severe MR (2.3%). Compared to pre-interventional MR grades, a significant reduction was achieved (*p* < 0.001) by TEER (Figure 2).

Depicted data comprises mitral regurgitation grades of all 86 patients at all time points: “pre”: Prior TEER, “post”: After TEER prior hospital discharge, “follow-up”: At the time of the last available implanted electrical cardiac device control. TEER: Transcatheter Edge-to-Edge Mitral Valve Repair.

In this cohort with severely depressed systolic LV function, improvements of MR grades were not associated with a significant change of LVEF (22.1 ± 10.3% to 23.6 ± 11.9%; *p* = 0.161).

In a univariate regression analysis change of LVEF (*p* = 0.341) and MR grade (*p* = 0.561) did not correlate with change of VA burden.

**Figure 3:**
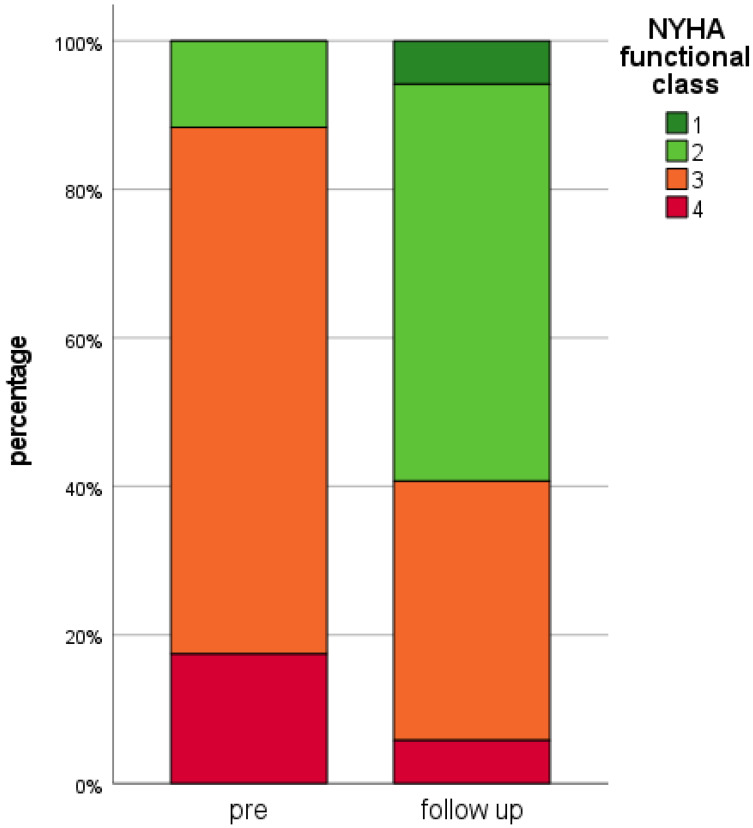
NYHA functional class at baseline and follow-up.

At follow-up, a significant clinical benefit according to NYHA functional class (*p* < 0.001) was observed. Median NYHA class was 2.0 (2.0; 3.0) with 51 patients (59.3%) presenting in NYHA functional class <3 after successful TEER (Figure 3).

Depicted data comprises NYHA functional class of all 86 patients prior to TEER (“pre”) and after TEER at the time of the last available implanted electrical cardiac device control (“follow-up”). NYHA: New York Heart Association, TEER: Transcatheter Edge-to-Edge Mitral Valve Repair.

**Figure 4:**
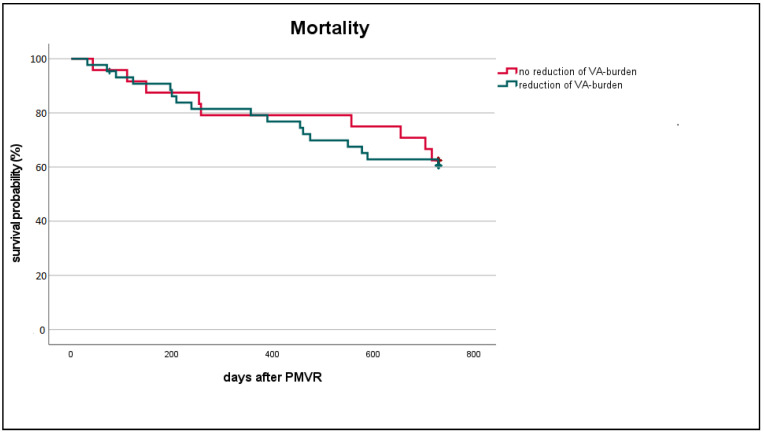
Kaplan-Meier curve for survival according to VA burden reduction. VA, ventricular arrhythmia; PMVR, percutaneous mitral valve repair; Log-Rank-Test: *p* = 0.815.

One-year mortality rate was 21.2% (18/85, information for one patient missing), two-year mortality rate was 38.6% (32/83, information for three patients missing). All-cause as well as cardiovascular mortality rates observed in patients with reduced VA burden (all-cause mortality: 38.6%, 17/44) did not significantly differ compared to patients without VA burden reduction (all-cause mortality: 37.5%, 9/24) (Log-Rank test for all-cause mortality *p* = 0.815, Figure 4; Supplementary Figure S1; Supplementary Table S2).

Applying univariate as well as multivariate adjusted regression analysis (using LVESD, LVEDD, NYHA functional class, ischaemic cardiomyopathy, baseline LVEF, age and gender as covariates) also revealed no significant mortality difference in patients with VA burden reduction compared to patients without VA burden reduction (univariate: *p* = 0.43; Hazard ratio (HR) 1.33 (95% confidence interval (CI) 0.66–2.66); multivariate: *p* = 0.84; HR 1.09 (95% CI 0.48–2.47)).

## 4. Discussion

The present study investigated the impact of TEER using the MitraClip^TM^ device on ventricular arrhythmias and ICD therapies. To date, only limited information regarding the effect of TEER on VA burden has been reported [13,14,15]. To the best of our knowledge, our study provides the longest follow-up information on ventricular arrhythmias after MitraClip^TM^ therapy published so far.

The key finding of our study is a significant reduction of VA burden and appropriate ICD therapies after TEER. Patients in whom TEER resulted in a reduced VA burden were in higher NYHA functional class, less frequently suffered from ischaemic cardiomyopathy and had larger LV dimensions prior to TEER. Successful MitraClip^TM^ therapy resulted in sustained mid-term MR reduction and clinical improvement according to NYHA functional class. However, reduction of VA burden did not result in improved two-year survival in this cohort with severely depressed LVEF.

The patients included in our study correspond to a real-world TEER-cohort with mainly functional MR due to cardiac remodelling in the context of advanced HF and thus very high surgical risk. The severely reduced LVEF and advanced LV dilatation of this cohort distinguishes our results from previous studies where less severe HF patients were included [13,14,15]. In addition, this cohort comprises a relevant percentage of patients with end-stage HF enlisted for cardiac transplantation, treated with MitraClip^TM^ as bridge therapy [21,22]. In total 21% (18/86) of the patients were listed for heart transplantation with a median age of 53 years, lowering the median age of the cohort comparing to earlier reports [13,14,15].

The VA data set of this cohort delivers robust and long-term data on VA burden and device therapy.

Safety and efficacy of MitraClip™ therapy in patients with severely impaired LVEF has already been demonstrated and TEER procedural failure has been identified as the most important predictor of one-year mortality in this cohort [12,23].

The prevalence of ventricular arrhythmias in our study population was high and, in the context of advanced HF with severely reduced LVEF, more pronounced compared with previously published cohorts [13,14,15]. As VA incidence correlates with LVEF, a potential mechanism of VA reduction by TEER could be due to LVEF improvement. However, LVEF was not significantly altered in this cohort nor in previously reported RCTs [12,24]. Therefore, we believe LVEF per se is not a comprehensive factor of VA reduction.

Further possible pathophysiological mechanisms triggering VA episodes comprise: (1) Volume overload of the LV as a result of severe MR causes increased myocardial wall stress and progressive LV remodelling [1] and fibrosis [25], which are known to correlate with the risk of ventricular arrhythmias [26,27,28]. (2) Tethering forces of the leaflets by LV enlargement and papillary muscle displacement causing mechanical irritation [29].

An observational study has shown a significant MR reduction after TEER, resulting in a significant increase in cardiac output and a significant reduction in LV filling pressures and pulmonary hypertension in patients with end-stage HF [21]. Thus, LV unloading following TEER-associated MR reduction may be an underlying mechanism for the significant decrease of VA burden and need for ICD therapies. In line with this hypothesis, Mierke et al. could recently show that in patients with cardiogenic shock a percutaneous LV assist device leads to a significant reduction of severe arrhythmias [30].

Analysis of pre-interventional clinical characteristics of patients showing a reduction of VA burden post TEER revealed higher NYHA functional class, non-ischaemic cardiomyopathy and larger left ventricular dimensions as significant parameters.

In line with our findings, advanced NYHA functional class has been previously shown to be an independent predictor for the occurrence of ventricular arrhythmias [31], sudden cardiac death and appropriate ICD therapies in patients with severely impaired LVEF [32]. Our findings are in line with previous studies with shorter follow-up. Thus, Benito-Gonzalez et al. found a trend towards more VA events in patients who presented in NYHA functional class IV prior to TEER [15]. Ledwoch et al. reported a better response of TEER in patients with dilated (DCMP) versus ischaemic cardiomyopathy as to the elimination of nsVT and/or sVT at 6-months follow-up (6/10 vs. 3/7, *p* = 0.006) [14]. In line with this, moderate to severe MR has previously been shown to be independently associated with the incidence of ventricular arrhythmias in DCMP patients [33] and larger LV dimensions have been described as predictors of serious arrhythmic events and appropriate ICD-shocks in patients presenting with non-ischaemic cardiomyopathy [34,35].

VA burden is related to worsening HF and is a predictor of sudden cardiac death in patients with systolic LV dysfunction [36]. Although we could observe a significant reduction of VA burden following TEER, this did not result in improved survival. One-year mortality rate in our study was comparable to previously published numbers from randomised trials (COAPT: 19.1%; Mitra-FR 24.2%) [12,24]. However, comparability with our study is limited as these two trials exclusively included HF patients presenting with functional MR (81.4% in our study) and severity of LVEF reduction was not as pronounced as in our study (mean LVEF COAPT: 31.3 ± 9.1%; Mitra-FR: 33.3 ± 6.5%). In comparison to registries including patients with both, functional and degenerative MR, one-year mortality in our study was higher (TRAMI: 20.3%; GIOTTO: 17.5%; ACCESS-EU: 17.3%; GRASP-IT: 15.1%; Sentinel-Pilot: 15.3%) [37,38,39,40,41]. However, our patient cohort presented with the most severe LVEF reduction, presumably representing the determining factor for the observed excess mortality. In line with this assumption, sub-group analyses of the TRAMI registry in patients with severely reduced LV-function comparable to our cohort revealed even higher one-year mortality rates (LVEF < 30%: 24.2%) [23].

The observed two-year mortality rate was in the range of the recently published expanded follow-up data of the Mitra-FR study (34.9%), the GIOTTO registry (34.6%) and the TRAMI registry (31.9%) [38,42,43] and higher in comparison to the COAPT trial (29.1%) and the GRASP-IT registry (26.4%) (data on two-year mortality of the other registries not available) [12,40].

The failure to demonstrate the association of reduced VA burden with improved survival may be due to the limited number of patients studied. In addition, it is well known that in advanced HF with severely impaired LVEF and cardiac remodelling, HF per se and not arrhythmias may be the predominant cause of death. However, the contrasting results of the COAPT- and Mitra-FR studies regarding a mortality benefit of MitraClip^TM^ therapy in addition to GDMT suggest a more complex explanation of this observation [44]. Baseline characteristics of the patients included in our study are more similar to the Mitra-FR cohort. In particular, our patients presented with more severe LV dilatation (LVEDD 68.2 mm; COAPT: 62 mm; Mitra-FR: 69 mm) compared to the COAPT cohort. In addition, they were in a more advanced stage of HF, which is also true when compared with the Mitra-FR cohort. In this context, our patients presented with a significantly lower LVEF (22.1 ± 10.3%; COAPT: 31.3 ± 9.1%; Mitra-FR: 33.3 ± 6.5%) prior to TEER. The more advanced stage of the underlying cardiac disease is also reflected in the proportion of severely symptomatic patients (88.4% NYHA class III/IV; COAPT: 57%; Mitra-FR: 63.1%) and the more pronounced elevation of NT-proBNP values (median 4567 ng/l (IQR 1957; 10,630 ng/l), mean 9392 ng/l ± 12,465 ng/l; COAPT: mean 5174 ng/l ± 6566 ng/l; Mitra-FR: median 3407 ng/l (IQR 1948–6790 ng/l)).

Consequently, one might conclude that in this severely ill patient cohort the progression of the underlying cardiac disease as well as the multitude of comorbidities are the predominant limitations of life expectancy rather than malignant rhythm disorders.

In conclusion, to avoid futility, future candidate selection for TEER should focus on patients with severe MR despite GDMT (including CRT) without too advanced adverse LV remodelling and dilatation, as recently published in the adjusted guideline recommendations based on the results of COAPT and Mitra-FR [10]. However, apart from the missing survival benefit, a significantly reduced VA burden, resulting in a decreased number of ICD therapies undoubtedly contributes to patients’ quality of life [45]. In this context, data of the German TRAMI registry already demonstrated distinctively improved quality of life after MitraClip^TM^ treatment in severe HF patients presenting with LVEF < 30% prior TEER [23]. Thus, on the basis of an individual case decision within the heart team, patients presenting with advanced HF, severely reduced LVEF and pronounced LV dilatation might also benefit from TEER.

The results of our study in conjunction with previously published data on this issue [13,14,15] should be considered “hypothesis-generating”. For a more thorough and valid analysis of VA reduction after TEER using the MitraClip^TM^ device, and possibly arising effects on mortality rates in this severely diseased patient group, a randomised clinical trial is mandatory.

### Limitations of the Study

The data were retrieved from a single centre in a retrospective approach. Although the completeness of the data is high and the follow-up period is comparatively long, the total number of studied patients is rather low. A potential selection bias due to a high proportion of patients enlisted for heart transplantation, thus providing more cardiac device Holter information in the context of frequent follow-up visits, is likely. In addition, the lack of an external core lab adjudicating the events may bias data interpretation. Changes in the dose of antiarrhythmic drugs, heart failure medication and in device programming as a result of a documented VA as well as the incidence of new ischaemic coronary events or revascularization procedures and potential metabolic disturbances were not addressed in this analysis. Furthermore, in this study 1st to 3rd generation MitraClip^TM^ was implanted. Meanwhile, 4th generation MitraClip^TM^ and the Pascal and Pascal Ace devices (Edwards Lifesciences, Irvine, CA, USA) have been FDA-approved and CE-certified for TEER.

## 5. Conclusions

The results of our study suggest that TEER results in a significant reduction of ventricular arrhythmias and ICD therapies in advanced HF patients. These effects were more prominent in patients presenting with non-ischaemic cardiomyopathy, higher baseline NYHA functional class and larger LV dimensions. While TEER did reduce VA burden, it did not result in improved two-year survival in this advanced HF cohort.

## Figures and Tables

**Table 1:** Baseline characteristics (*n* = 86).

Characteristics		Value
Sex (male)		69/86 (80.2%)
Age (years; median)		66.5 [58; 76]
mitral regurgitation etiology	degenerative	6/86 (7.0%)
	functional	70/86 (81.4%)
	mixed	10/86 (11.6%)
Left ventricular ejection fraction (%)		22.1 (±10.3)
Left ventricular ejection fraction ≤ 35%		78/86 (90.7%)
LA Diameter (mm)		54.6 (±9.7)
LVESD (mm)		59.0 (±12.3)
LVEDD (mm)		68.2 (±11.0)
Systolic PA pressure (TTE; mmHg)		51 (±13)
hsTNT (pg/mL)		45.0 (±39.2)
NT-proBNP (median; ng/l) ^#^		4567 [1957; 10,630]
6 min walk test distance (m) ^#^		335 (±126)
NYHA stage (mean)		3.1 (±0.5)
Stage 1		0/86 (0%)
Stage 2		10/86 (11.6%)
Stage 3		61/86 (70.9%)
Stage 4		15/86 (17.4%)
STS Score (median; %)		5.0 [2.5; 9.7]
EuroScore II (%)		12.5 (±12.0)
Significant CAD		63/86 (73.3%)
Prior cardiothoracic surgery		33/86 (38.4%)
Atrial fibrillation		56/86 (65.1%)
Prior stroke		7/86 (8.1%)
Increased retention values (Creatinine ≥ 1.3 mg/dL)		45/86 (52.3%)
Sleep apnoea syndrome		6/86 (7.0%)
Pulmonary disease		16/86 (18.6%)
Diabetes mellitus		23/86 (26.7%)
Cancer	active	6/86 (7.0%)
	state after	3/86 (3.5%)
Implantable cardioverter defibrillator (ICD)		42/86 (48.8%)
Cardiac resynchronization therapy—pacemaker (CRT-P)		1/86 (1.2%)
Cardiac resynchronization therapy—defibrillator (CRT-D)		39/86 (45.3%)
Pacemaker		4/86 (4.7%)
Prior ventricular arrhythmia *	nsVT	50/86 (58.1%)
	sustained VT	36/86 (41.9%)
	VF	11/86 (12.8%)
	any sustained VA	40/86 (46.5%)
	any VA	61/86 (70.9%)
Prior Cardiopulmonary Resuscitation Patients enlisted for heart transplantation		9/86 (11.6%) 18/86 (20.9%)

LA, left atrium; LVESD, left ventricular end-systolic diameter; LVEDD, left ventricular end-systolic diameter; PA, pulmonary artery; TTE, transthoracic echocardiography; hsTNT, high-sensitive Troponin T; NT-proBNP, N-terminal prohormone Brain Natriuretic Peptide; NYHA, New York Heart Association; STS, Society of Thoracic Surgeons; CAD, coronary artery disease; nsVT, non-sustained ventricular tachycardia; VT, ventricular tachycardia; VF, ventricular fibrillation; VA, ventricular arrhythmia. ^#^ Values are available of 83/86 patients for NT-proBNP and 63/86 patients for 6 min walk test distance * during the study period.

**Table 2:** Ventricular arrhythmias and ICD therapies before and after percutaneous mitral valve repair (events per patient per month, *n* = 86).

Ventricular Arrhythmic Event	Before TEER	After TEER	*p*-Value
Any ventricular arrythmia	2.24 ± 5.09	1.26 ± 3.52	0.019
Non-sustained ventricular tachycardia	1.39 ± 3.31	0.83 ± 2.09	0.120
Any sustained ventricular arrhythmia	0.85 ± 3.47	0.43 ± 2.03	0.010
Sustained ventricular tachycardia	0.82 ± 3.46	0.43 ± 2.03	0.014
Ventricular fibrillation	0.035 ± 0.186	0.005 ± 0.033	0.056
Any appropriate device-therapy *	1.00 ± 3.87	0.32 ± 1.41	0.014
Appropriate antitachycardia pacing *	0.82 ± 3.56	0.28 ± 1.31	0.008
Appropriate ICD shocks *	0.18 ± 0.95	0.04 ± 0.12	0.052
Left ventricular function			
LVEF (%)	22.1 ± 10.3	23.6 ± 11.9	0.161

* Among patients with ICD or CRT-D. TEER, transcatheter mitral valve edge-to-edge repair; ICD, Implantable Cardioverter Defibrillator; CRT-D, Cardiac Resynchronization Therapy—Defibrillator; LVEF, left ventricular ejection fraction.

**Table 3:** Patient characteristics according to presence of ventricular arrhythmias.

All Patients (*n* = 68)		Reduction of Ventricular Arrhythmia Burden (*n* = 44)	No Reduction of Ventricular Arrhythmia Burden (*n* = 24)	*p*-Value
Sex (male)	56/68 (82.4%)	39/44 (88.6%)	17/24 (70.8%)	0.096
Age (years)	65.7 ± 12.3	68.1 ± 11.7	64.2 ± 12.5	0.319
Ejection fraction (%)	21.6 ± 9.7	21.3 ± 10.3	22.3 ± 8.6	0.292
LA Diameter (mm)	54.8 ± 10.4	56.0 ± 11.3	52.6 ± 8.4	0.452
LVESD (mm)	59.0 ± 12.0	61.1 ± 12.0	55.4 ± 11.4	0.043
LVEDD (mm)	68.3 ± 10.8	70.3 ± 10.6	64.6 ± 10.4	0.037
Systolic PA pressure (TTE; mmHg)	50 ± 13	52 ± 13	48 ± 13	0.306
hsTNT (pg/mL)	45.1 ± 39.0	49.3 ± 44.5	37.3 ± 25.1	0.390
NT-proBNP (ng/l)	8559 ± 10,974	8455 ± 8339	8742 ± 14,708	0.305
6 min walk test distance (m)	338 ± 121	327 ± 127	363 ± 103	0.340
NYHA stage	3.1 ± 0.6	3.2 ± 0.5	2.8 ± 0.6	0.004
STS Score (%)	7.7 ± 7.8	7.1 ± 6.9	8.9 ± 9.3	0.529
EuroScore II (%)	12.7 ± 12.3	13.1 ± 12.9	11.9 ± 11.4	0.488
Significant CAD	49/68 (72.1%)	28/44 (63.6%)	21/24 (87.5%)	0.048
Prior cardiothoracic surgery	26/68 (38.2%)	16/44 (36.4%)	10/24 (41.7%)	0.667
Atrial fibrillation	43/68 (63.2%)	27/44 (61.4%)	16/24 (66.7%)	0.665
Prior stroke	6/68 (8.8%)	3/44 (6.8%)	3/24 (12.5%)	0.658
Increased retention values (Creatinine ≥ 1.3 mg/dL)	36/68 (52.9%)	24/44 (54.5%)	12/24 (50.0%)	0.720
Sleep apnoea syndrome	6/68 (8.8%)	2/44 (4.5%)	4/24 (16.7%)	0.175
Pulmonary disease	12/68 (17.6%)	10/44 (22.7%)	2/24 (8.3%)	0.190
Diabetes mellitus	19/68 (27.9%)	14/44 (31.8%)	5/24 (20.8%)	0.335
Cancer (‘state after’ included)	5/68 (7.4%)	2/44 (4.5%)	3/24 (12.5%)	0.337

Patients with no occurrence of ventricular arrhythmia during the entire study period were excluded (*n* = 18). LA, left atrium; LVESD, left ventricular end-systolic diameter; LVEDD, left ventricular end-diastolic diameter; PA, pulmonary artery; TTE, transthoracic echocardiography; hsTNT, high-sensitive Troponin T; NT-proBNP, N-terminal prohormone Brain Natriuretic Peptide; NYHA, New York Heart Association; STS, Society of Thoracic Surgeons; CAD, coronary artery disease.

## Data Availability

Not applicable.

## Supplementary Materials

The following supporting information can be downloaded at: https://www.mdpi.com/article/10.3390/life12030344/s1, Figure S1: Kaplan-Meier curve for cardiovascular mortality according to VA burden reduction; Table S1: Baseline and procedural characteristics of excluded patients; Table S2: Mortality reasons.

## Abbreviations

ATP	Anti-tachycardia Pacing
CI	Confidence Interval
CRT	Cardiac Resynchronization Therapy
CRT-D	Cardiac Resynchronization Therapy—Defibrillator
CRT-P	Cardiac Resynchronization Therapy—Pacemaker
DCMP	Dilated Cardiomyopathy
GDMT	Guideline-Directed Medical Therapy
HF	Heart Failure
HFrEF	Heart Failure with reduced Ejection Fraction
HR	Hazard Ratio
ICD	Implantable Cardioverter Defibrillator
LA	Left Atrial
LV	Left Ventricular
LVEDD	Left Ventricular Enddiastolic Diameter
LVESD	Left Ventricular Endsystolic Diameter
LVEF	Left Ventricular Ejection Fraction
MR	Mitral Regurgitation
nsVT	non-sustained Ventricular Tachycardia
NT-proBNP	N-terminal prohormone Brain Natriuretic Peptide
NYHA	New York Heart Association
sVT	sustained Ventricular Tachycardia
TEER	Transcatheter Edge-to-Edge Repair
VA	Ventricular Arrhythmia
VF	Ventricular Fibrillation
VT	Ventricular Tachycardia
