# Supplementary material for: Impact of Percutaneous Mitral Valve Repair Using the MitraClipTM System on Ventricular Arrhythmias and ICD Therapies
Source: Life (Basel). 2022 Feb 25;12(3):344. doi: 10.3390/life12030344 (PMC8950873; doi:10.3390/life12030344)
Supplement: Supplementary file 1 [file life-12-00344-s001.zip › life-1613463-supplementary.pdf]

life

MDPI

Supplementary Materials

Impact of Percutaneous Mitral Valve Repair Using the MitraClip™ System on Ventricular Arrhythmias and ICD Therapies

Nicolas A. Geis *, Anna Göbbel, Michael M. Kreusser, Tobias Täger, Hugo A. Katus, Norbert Frey, Philipp Schlegel † and Philip W. Raake †

Department of Internal Medicine III, University of Heidelberg, Im Neuenheimer Feld 410, 69120 Heidelberg, Germany; anna-goebbel@web.de (A.G.); michael.kreusser@med.uni-heidelberg.de (M.M.K.); tobias.taeger@med.uni-heidelberg.de (T.T.); hugo.katus@med.uni-heidelberg.de (H.A.K.); norbert.frey@med.uni-heidelberg.de (N.F.); philipp.schlegel@med.uni-heidelberg.de (P.S.); philip.raake@med.uni-heidelberg.de (P.W.R.)

* Correspondence: nicolas.geis@med.uni-heidelberg.de; Tel.: +49-6221-56-8676; Fax: +49-6221-56-5515

† These authors contributed equally to this manuscript.

Table S1. Baseline and procedural characteristics

**Baseline characteristics**		Patients included in the study (n=86)	Patients excluded due to missing cardiac device holter information (n=167)	p-value
Sex (male)		69/86 (80.2%)	127/167 (76.0%)	0.450
Age (years; median)		66.5 [58;76]	72.0 [61;82]	<0.001
mitral regurgitation etiology	degenerative	6/86 (7.0%)	24/162 (14.8%)	
	functional	70/86 (81.4%)	110/162 (67.9%)	0.066
	mixed	10/86 (11.6%)	28/162 (17.3%)	
Left ventricular ejection fraction (%)		22.1 (±10.3)	27.7 (±13.7)	0.008
Left ventricular ejection fraction ≤35%		78/86 (90.7%)	123/167 (73.7%)	0.003
LA Diameter (mm)		54.6 (±9.7)	51.6 (±6.6)	0.006
LVESD (mm)		59.0 (±12.3)	52.0 (±13.5)	<0.001
LVEDD (mm)		68.2 (±11.0)	63.3 (±10.8)	<0.001
Systolic PA pressure (TTE; mmHg)		51 (±13)	50 (±13)	0.686
hsTNT (pg/ml)		45.0 (±39.2)	45,1 (±34.2)	0.282
NT-proBNP (ng/l)		9392 (±12465)	8274 (±10767)	0.232
6 minute walk test distance (m)		335 (±126)	305 (±151)	0.206
NYHA stage (mean)		3.1 (±0.5)	3.2 (±0.5)	**0.017**
Stage 1		0/86 (0%)	0/167 (0%)	
Stage 2		10/86 (11.6%)	9/167 (5.4%)	
Stage 3		61/86 (70.9%)	110/167 (65.9%)	
Stage 4		15/86 (17.4%)	48/167 (28.7%)	
EuroScore II (%)		12.5 (±12.0)	9.8 (±8.5)	0.089
Significant CAD		63/86 (73.3%)	84/167 (50.3%)	**0.020**
Prior cardiothoracic surgery		33/86 (38.4%)	45/167 (26.6%)	0.062
Atrial fibrillation		56/86 (65.1%)	107/167 (64.1%)	0.869
Prior stroke		7/86 (8.1%)	24/167 (14.4%)	0.152
Increased retention values (Creatinine ≥ 1.3 mg/dL)		45/86 (52.3%)	95/167 (56.9%)	0.489

Sleep apnoea syndrome	6/86 (7.0%)	9/167 (5.4%)	0.613
Pulmonary disease	16/86 (18.6%)	32/167 (19.2%)	0.915
Diabetes mellitus	23/86 (26.7%)	63/167 (37.7%)	0.081
Cancer — active	6/86 (7.0%)	6/167 (3.6%)	0.471
Cancer — state after	3/86 (3.5%)	5/167 (3.0%)	
Implantable cardioverter defibrillator (ICD)	42/86 (48.8%)	84/167 (50.3%)	0.826
Cardiac resynchronization therapy - pacemaker (CRT-P)	1/86 (1.2%)	3/167 (1.8%)	1.000
Cardiac resynchronization therapy - defibrillator (CRT-D)	39/86 (45.3%)	37/167 (22.2%)	**0.001**
Pacemaker	4/86 (4.7%)	41/167 (24.6%)	**0.001**
Prior Cardiopulmonary Resuscitation	9/86 (11.6%)	10/167 (6.0%)	0.201

Procedural characteristics

Number of implanted MitraClip™ devices (mean)	1.37 (±0.51)	1.41 (±0.56)	0.712
MR grade preprocedural (mean)	3.0 (±0.05)	3.0 (±0.00)	0.340
MR grade postprocedural (mean)	1.15 (±0.47)	1.20 (±0.54)	0.643
Technical success	86/86 (100%)	167/167 (100%)	1.000
Device success	84/86 (97.7%)	162/167 (97.0%)	1.000

LA, left atrium; LVESD, left ventricular end-systolic diameter; LVEDD, left ventricular end-diastolic diameter; PA, pulmonary artery; TTE, transthoracic echocardiography; hsTNT, high-sensitive Troponin T; MR, mitral regurgitation; NT-proBNP, N-terminal prohormone Brain Natriuretic Peptide; NYHA, New York Heart Association; STS, Society of Thoracic Surgeons; CAD, coronary artery disease; nsVT, non-sustained ventricular tachycardia; VT, ventricular tachycardia; VF, ventricular fibrillation; VA, ventricular arrhythmia .

Table S2. Mortality reasons

2-year Mortality	Value
All-cause	32/83 (38.6%)
- Cardiovascular	6/32 (18.8%)
- Non-cardiovascular	9/32 (28.1%)
- Unknown reason	17/32 (53.1%)

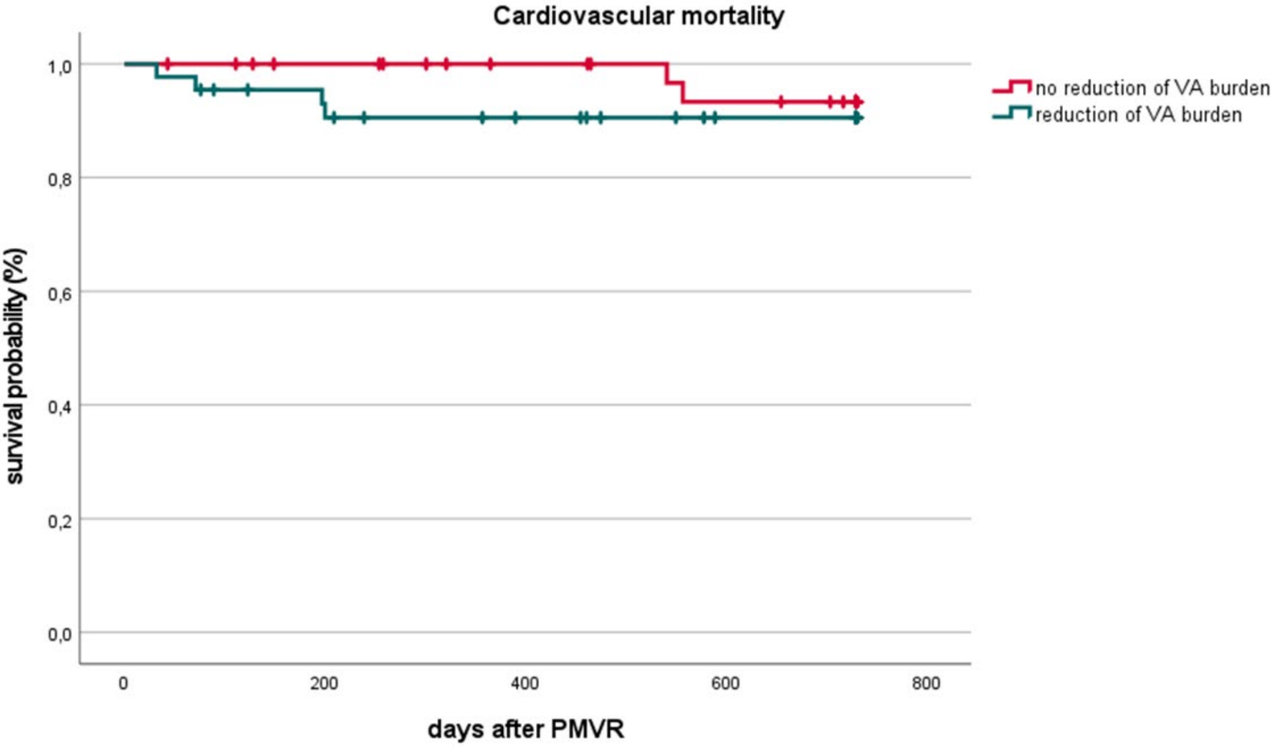

Figure S1. Kaplan-Meier curve for cardiovascular mortality according to VA burden reduction. VA, ventricular arrhythmia; PMVR, percutaneous mitral valve repair; Log-Rank-Test: p=0.441.
